# Prize Dissertations

**Published:** 1850-07

**Authors:** 


*Prize Dissertations.* By USHER PARSONS, M. D., late Prof. of Anatomy and Surgery, Brown University, and Surgeon U. S. Navy, etc., etc. Second Edition. Providence: printed by T. Albro, 1849. 8 vo. pp. 248.

This volume, published some months since, contains several dissertations which gained the Boylston prizes annually awarded to the successful competitors for the best essays on a stated subject. The author's uniform and successive triumphs in this way, had become so much a matter of course, that, some years ago, when the subjects were announced for the coming year, the Boston Medical and Surgical Journal stated, as an inducement for medical writers to enter the list, that Dr. Parsons had engaged not to engage in the contest.

The Boylston prize fund was instituted in 1803, by Ward Nicholas Boylston, a descendant of Zabdiel Boylston, F. R. S., who introduced into America the practice of inoculation for small pox in 1721. The fund provides a perpetual income of $100, which is divided between the authors of the best dissertations on two different subjects given out two years before, the successful candidates receiving either the money, or a gold medal of the same value, at their option. The management of the fund, and the adjudication of prizes, are entrusted to a committee. This committee consists of not less than seven, nor more than eleven members, who receive their appointment from the President and fellows of Harvard University.

Competition for the prizes is invited from members of the profession throughout the United States, not being confined, as is the course pursued in some States, to the medical men within the State. These facts we gather from the preface of Dr. Parsons' work.

The subjects of the dissertations which the volume contains, are:

1. On inflammation of the Periosteum, both acute and chronic. Premium awarded in 1827.

2. On the disease called an irritable state of the urinary bladder; its causes and treatment. Premium awarded in 1828.

3. On the connection between cutaneous diseases which are not contagious, and the internal organs. Premium awarded in 1830.

4. On the diagnostic marks of cancer of the breast; and whether it be curable. Premium awarded in 1835.

5. On the comparative influence of Malaria as a cause of Fever. Written 1830.

The subjects of these dissertations are interesting, and practically important. The author, as it is gratuitous to remark, is a gentleman of large experience, particularly in the department of surgery, deeply versed in the literature of the science, a close thinker, and a judicious practitioner. At the meeting of the American Medical Association in May, 1849, he was appointed chairman of the Committee on Medical Science, and his report in the forth coming volume of transactions, will, doubtless, furnish abundant evidence of the propriety of the selection. In reproducing these dissertations in a form which will ensure their perpetuity, Dr. Parsons has discharged a duty to himself, while he has conferred a benefit on medical literature.

